# S1‐Leitlinie Diagnostik und Therapie der Necrobiosis lipoidica

**DOI:** 10.1111/ddg.15943_g

**Published:** 2026-03-09

**Authors:** Cornelia Erfurt‐Berge, Regina Renner, Melanie Peckruhn, Jörg Tittelbach, Dorothea Terhorst‐Molawi, Friederike Kauer, Joachim Dissemond

**Affiliations:** ^1^ Hautklinik Uniklinikum Erlangen, Friedrich‐Alexander‐Universität Erlangen‐Nürnberg; ^2^ Praxis Esslingen; ^3^ Klinik für Hautkrankheiten Universitätsklinikum Jena Friedrich‐Schiller‐Universität Jena; ^4^ Institut für Allergieforschung Charité – Universitätsmedizin Berlin; ^5^ Fraunhofer‐Institut für Translationale Medizin und Pharmakologie (ITMP) Immunologie und Allergologie, Berlin; ^6^ Praxis Potsdam; ^7^ Klinik und Poliklinik für Dermatologie Venerologie und Allergologie Universitätsklinikum Essen

**Keywords:** Diabetes mellitus, Lebensqualität, Necrobiosis lipoidica, S1‐Leitlinie, Ulcus cruris, Diabetes mellitus, leg ulcer, necrobiosis lipoidica, quality of life, S1 guideline

## Abstract

Die Necrobiosis lipoidica (NL) ist eine seltene granulomatöse Hauterkrankung unklarer Ätiologie, die gehäuft assoziiert mit Diabetes mellitus und anderen Komorbiditäten auftreten kann. Prädilektionsstellen sind die Unterschenkel, und hier insbesondere die prätibialen Bereiche. Die exakte Pathogenese ist weiterhin unklar. Es werden vaskuläre Störungen mit mikroangiopathischen Veränderungen und eine autoimmunologische Genese diskutiert. Die Necrobiosis lipoidica tritt drei‐ bis sechsmal häufiger bei Frauen auf. Männer zeigen eher einen schwereren Verlauf und entwickeln häufiger Ulzerationen. Die Diagnose kann oft aufgrund der typischen klinischen und dermatoskopischen Befunde gestellt werden. Biopsien sollten in klinisch unklaren Fällen, bei Ulzeration oder bei Hinweisen auf maligne Entartung erfolgen. Insgesamt ist die wissenschaftliche Datenlage für die NL noch unzureichend und es besteht weiterer Forschungsbedarf. Da die Patienten oft eine starke Einschränkung der Lebensqualität haben, ist es wichtig die wenigen wissenschaftlichen Erkenntnisse zu kennen und in entsprechende praxisrelevante Therapieempfehlungen umzusetzen. Diese Kurzversion der S1‐Leitlinie der Deutschen Dermatologischen Gesellschaft (DDG) fasst die aktuellen Erkenntnisse zusammen und gibt unter Einbeziehung von Experteneinschätzungen konkrete Handlungsempfehlungen für den klinischen Alltag.

## EINLEITUNG

Die Necrobiosis lipoidica (NL) ist eine seltene granulomatöse Hauterkrankung unklarer Ätiologie (Orphanet Code: 542592). Verlässliche Angaben zu Prävalenz und Inzidenz gibt es nicht. Bei Patienten mit diabetesassoziierter NL liegt das durchschnittliche Erkrankungsalter in der 3. und 4. Lebensdekade; bei nicht diabetesassoziierter NL ist das durchschnittliche Erkrankungsalter die 4. und 5. Lebensdekade. Patienten mit Typ‐1‐Diabetes erkranken meist in jüngerem Alter.^1^, ^2^ Die Necrobiosis lipoidica tritt bei Frauen drei‐ bis sechsmal häufiger als bei Männern auf; Männer zeigen häufiger schwerere Verläufe und entwickeln öfter Ulzerationen.^3^, ^4^, ^5^, ^6^

## PATHOGENESE

Die genaue Pathogenese der NL ist weiterhin unklar. Es werden vaskuläre Störungen mit mikroangiopathischen Veränderungen mit Immunkomplexablagerungen sowie ausgeprägte Kollagendegenerationen im Rahmen einer autoimmunologischen Genese diskutiert.^7^, ^8^ Traumata sowie metabolische und entzündliche Prozesse führen zu einer Aktivierung und Migration der Neutrophilen, die das entzündliche Infiltrat im Frühstadium der Erkrankung dominieren.^9^ Die durch die Neutrophilen bedingte Makrophagenproliferation resultiert in der Bildung von Granulomen. Die genetische Disposition in der Ätiopathogenese ist unklar; es gibt aber mehrere Berichte über familiäre Fälle von NL, die mit oder ohne Diabetes mellitus assoziiert sein können.^9^, ^10^, ^11^, ^12^ Die häufigsten pathologischen vaskulären Veränderungen der NL sind Verdickung der Gefäßwände, Fibrose und endotheliale Proliferation.^13^ In der Immunfluoreszenzdiagnostik konnten Ablagerungen von Fibrin, Fibrinogen, Immunglobulinen (vor allem IgM) und Komplementfaktoren (vor allem C3) an der dermoepidermalen Grenzfläche der Blutgefäße nachgewiesen werden.^14^ Die Gefäßverdickungen und die daraus resultierende erhöhte Aktivierung des Gerinnungssystems werden durch Glykoproteinablagerungen verursacht, die in den Gefäßwänden von Menschen mit Diabetes und Patienten mit NL gefunden werden.^15^, ^16^ Ein vermehrtes Vorkommen von Glut‐1‐Rezeptoren (humaner Erythrozyten‐Glukosetransporter) in Fibroblasten wird als möglicher Faktor diskutiert, der den Blutfluss beeinflusst.^17^ Die Konzentration von Kollagen ist bei der NL reduziert. Diskutiert werden erhöhte Lysyloxidase‐Spiegel, die für die Kollagenvernetzung bei Menschen mit Diabetes verantwortlich sind, was zu Endorganschäden, beschleunigter Alterung und auch zu der für NL typischen wachsartigen Verdickung der Gefäßmembranen führt.^18^

## DIAGNOSTIK UND KLINISCHER VERLAUF

Die Diagnose der Necrobiosis lipoidica kann häufig anhand des typischen klinischen Bildes gestellt werden. Eine sorgfältige Anamnese und gegebenenfalls serologische Untersuchungen erfassen den Krankheitsbeginn, die Ausbreitung und Komorbiditäten wie Diabetes mellitus sowie die Abgrenzung zu Differenzialdiagnosen. Das typische klinische Bild der NL sind ovale Plaques mit bräunlichen bis bräunlich‐lividen Rändern und einem zentral initial rötlich‐braunen und später gelblich‐bräunlichen atrophen Zentrum und Teleangiektasien.^19^ In der Dermatoskopie zeigen sich scharf begrenzte, verlängerte und stark geschlängelte Teleangiektasien auf einem weißlichen oder gelblich‐orangefarbenen, strukturlosen Hintergrund.^20^, ^21^, ^22^ Die meisten Patienten haben im frühen Stadium kaum klinische Symptome. Es können jedoch Juckreiz, Dysästhesie oder Schmerzen auftreten.^23^ Die Prädilektionsstelle sind die Unterschenkel, und hier insbesondere die prätibialen Bereiche.^1^, ^2^ In circa 7–15% wurden Hautveränderungen der Arme oder des Abdomens sowie der Genitalbereich beschrieben, meist als zusätzliche Läsionen.^1^, ^24^, ^25^, ^26^ Die Hautveränderungen können einzeln auftreten, sind aber häufiger multipel vorhanden.^27^ Der Verlauf der Erkrankung ist chronisch. Als Komplikation können bei bis zu 35% der Betroffenen Ulzerationen auftreten, häufig nach einer Bagatellverletzung.^3^, ^4^, ^28^ Risikofaktoren für Ulzerationen sind männliches Geschlecht und ein (unzureichend kontrollierter) Diabetes mellitus.^29^ Eine seltene Komplikation ist die Entwicklung kutaner Neoplasien insbesondere auf dem Boden einer ulzerierten NL nach meist jahrelangem Verlauf.^30^, ^31^, ^32^, ^33^ Für die Diagnostik der NL existieren keine spezifischen Laborparameter (Tabelle 1).

**TABELLE 1:** Empfehlungen zur Diagnostik der Necrobiosis lipoidica.

**Empfehlung**	**Stärke**
Für die Diagnose der Necrobiosis lipoidica *wird empfohlen*, folgende Aspekte zu berücksichtigen: *Anamnese* – Tritt häufiger bei Frauen als bei Männern auf – Typisches Auftreten im mittleren bis höheren Erwachsenenalter – Bei Typ‐1‐Diabetes sind auch Fälle im Kindesalter beschrieben – Vorliegen einer assoziierten Erkrankung *Klinik* – Bräunlich‐gelbliche Plaques mit initial entzündlichem Randsaum, im Verlauf atrophem Zentrum – Häufige Lokalisation an den Unterschenkeln, insbesondere prätibial – Auftreten einer Ulzeration in den Plaques möglich Eine dermatoskopische Untersuchung *kann* als ergänzende Diagnostik *erwogen werden*.	↑↑ 0
Für die Diagnostik einer Necrobiosis lipoidica *wird* die Entnahme einer Gewebeprobe zur histologischen Sicherung in klinisch unklaren Fällen, bei Ulzeration oder bei Hinweisen auf eine maligne Entartung *empfohlen*.	↑↑

↑↑ – wird empfohlen, ↑ – kann empfohlen werden, 0 – kann erwogen werden

↑↑ – is recommended, ↑ – may be recommended, 0 – may be considered

## HISTOLOGIE

Das histologische Bild ist variabel, abhängig von der Bestandsdauer und wird beeinflusst von einer Assoziation mit Diabetes mellitus.^34^ Unter einer meist unauffälligen Epidermis, die auch atroph, akanthotisch, von Hyperkeratose bedeckt und selten ulzeriert sein kann, zeigen sich bei Menschen mit Diabetes mellitus im gesamten Korium bis in die Subkutis und vor allem in die Bindegewebssepten reichend, Palisadengranulome und bei Menschen ohne Diabetes eher granulomatöse Reaktionen vom sarkoidalen Typ.^35^ In frühen Läsionen findet sich ein superfizielles und tiefes gemischtzelliges oder Neutrophilen‐betontes entzündliches Infiltrat, das teilweise in die Bindegewebssepten oder in das subkutane Fettgewebe reicht. Es können eine nekrotisierende Vaskulitis in der Nähe von Nekrobiosezonen und auch Nekrose von Adnexstrukturen entstehen. Die meist in horizontaler Ausrichtung linear angeordneten Nekrobiosezonen bestehen aus eosinophilen, geschwollenen oder degenerierten kollagenen Fasern, die hyalinisiert erscheinen und von Lymphozyten und Histiozyten umgeben sind. Plasmazellen sind in der Regel beigemengt. Mehrkernige Riesenzellen vom Langerhans‐ oder Fremdkörpertyp können zahlreich sein und dominieren in den Bindegewebssepten, vor allem beim sarkoidalen Typ. Die Nekrobiosezonen enthalten selten Muzin und weisen gleichzeitig einen Verlust von elastischen Fasern auf, was sich in der Elastica‐van‐Gieson‐Färbung darstellen lässt.^36^ Vaskuläre Veränderungen, wie Wandverdickung, Intimaproliferation und Lumeneinengung mit Thrombenbildung sind möglich. Bei länger bestehenden Läsionen ist das Bindegewebe parallelisiert, fibrosiert und die kollagenen Fasern sklerosiert mit eingestreuten Plasmazellen. Im Spätstadium findet sich ein Verlust der elastischen Fasern.^37^

Bei klinisch eindeutigen Befunden ist die Biopsie wegen des hohen Risikos für Wundheilungsstörungen verzichtbar.^29^

## DIFFERENZIALDIAGNOSEN

Differentialdiagnostisch müssen andere Erkrankungen mit ähnlichem klinischem oder histologischem Bild abgegrenzt werden.^38^ (Tabelle 2). Beispielsweise in Abgrenzung zum Granuloma anulare finden sich in der Dermatoskopie keine Teleangiektasien, sondern meist nur strukturlose orange bis rötliche periphere lineare Erytheme.^20^ Selten kann auch eine kutane Sarkoidose mit NL‐ähnlichen Hautveränderungen auftreten.^39^

**TABELLE 2:** Differenzialdiagnosen der Necrobiosis lipoidica.

Wichtige Differenzialdiagnosen der Necrobiosis lipoidica
Granuloma anulare
Kutane Sarkoidose
Nekrobiotisches Xanthogranulom
*Bei Ulzeration*:
Ulcus cruris venosum
Ulcus cruris arteriosum
Ulzerierte Neoplasie
Andere Ursachen eines Ulcus cruris

Seltenere Differentialdiagnosen
Morphea
Stauungsdermatitis, Purpura jaune d'ocre
Erythema nodosum
Erythema induratum
Lichen sclerosus et atrophicus
Radioderm
Tertiäre Lues
Noduläre Pannikulitis
Tuberkuloide Lepra

## ASSOZIIERTE ERKRANKUNGEN

Eine häufig assoziierte Erkrankung ist bei 11%–65% der Patienten mit NL ein Diabetes mellitus.^1^, ^3^, ^4^, ^40^ Die NL kann auch einer Diabetes‐Erkrankung vorangehen beziehungsweise zeitgleich diagnostiziert werden. Bei vielen Erkrankten, vor allem mit Typ‐1‐Diabetes, ist die Blutzuckereinstellung insuffizient.^1^, ^41^ Dabei tritt die NL bei weniger als 1% aller Menschen mit Diabetes mellitus auf.^42^, ^43^ Weitere assoziierte Erkrankungen sind Schilddrüsenfunktionsstörungen bei etwa 15%–25% der Patienten, vor allem Hypothyreose.^1^, ^29^ Es wird daher empfohlen, diese Erkrankungen bei NL‐Patienten labordiagnostisch zu kontrollieren. Es konnte gezeigt werden, dass es zu einer Besserung von NL‐Läsionen nach Optimierung der diabetischen Stoffwechsellage bei Patienten mit Typ‐1‐Diabetes kommt.^44^ Somit scheint es einen kausalen Zusammenhang zwischen glykämischer Stoffwechsellage und NL zu geben. In Anbetracht der generellen Auswirkungen des Diabetes, insbesondere auch auf die Wundheilung, ist eine Optimierung als sinnvoller, wenn auch nicht alleinstehender Faktor zu sehen. Zudem sind mit Adipositas, arteriellem Hypertonus und Fettstoffwechselstörungen weitere Aspekte des metabolischen Syndroms gehäuft assoziiert, wobei ein ursächlicher Zusammenhang bisher nicht eindeutig geklärt werden konnte.^4^, ^45^

## THERAPIE

### Topische Therapien

Topische Therapien (Tabelle 3) können im aktiven inflammatorischen Randbereich wirksam sein. Oftmals besteht im Zentrum der Hautveränderungen bereits eine Atrophie und bindegewebiger Umbau, ohne dass eine wesentliche Inflammation nachweisbar wäre. Daher ist die topische antientzündliche Therapie im Zentrum der Läsionen meist nicht erfolgversprechend. Hochpotente topische Glukokortikosteroide und topisches Tacrolimus (*off label*) werden am häufigsten verwendet und zeigen ein gutes Ansprechen.^4^ Eine kleine Zahl von Fallberichten gibt Hinweise auf eine mögliche Wirksamkeit der intraläsionalen Anwendung von Infliximab (*off label*),^46^, ^47^ ebenso wie der topischen Anwendung des JAK1‐ und JAK2‐Inhibitors Ruxolitinib (*off label*).^48^

**TABELLE 3:** Empfehlungen zur Lokaltherapie der Necrobiosis lipoidica.

**Empfehlung**	**Stärke**
Die Anwendung topischer Glukokortikosteroide, bevorzugt der Klasse III–IV nach Niedner, im Randbereich inflammatorischer Necrobiosis‐lipoidica‐Läsionen *wird* als primäre lokale Therapiemaßnahme *empfohlen*.	↑↑
Die Anwendung von Tacrolimus‐Salbe (*off label*) *wird* als Alternative zu topischen Glukokortikosteroiden zur antiinflammatorischen Lokaltherapie *empfohlen*.	↑↑
Die topische Anwendung von Ruxolitinib (*off label*) *kann erwogen werden*.	0
Eine intraläsionale Anwendung von Glukokortikosteroiden *kann* bei fehlendem Therapieansprechen *erwogen werden*.	0
Die intraläsionale Anwendung von Infliximab (*off label*) *kann* bei Versagen anderer Therapiemaßnahmen *erwogen werden*.	0

↑↑ – wird empfohlen, ↑ – kann empfohlen werden, 0 – kann erwogen werden

↑↑ – is recommended, ↑↑ – may be recommended, 0 – may be considered

### Glukokortikosteroide

Die topische Anwendung von Glukokortikosteroiden ist seit vielen Jahrzehnten der Therapiestandard.^4^, ^29^ Auch wenn keine prospektiven randomisierten, kontrollierten klinischen Studien existieren, die die Wirksamkeit von Glukokortikosteroiden bei NL nachweisen, besteht Expertenkonsens, dass initial eine Therapie mit topischen Glukokortikosteroiden versucht werden soll.^4^ Aufgrund des granulomatösen Charakters und der Tiefe des inflammatorischen Infiltrats sollten bevorzugt hochpotente Glukokortikosteroide, gegebenenfalls unter okklusiven Bedingungen, eingesetzt werden.^4^ Wenn auch unter okklusiver topischer Therapie mit Glukokortikosteroiden kein zufriedenstellender Therapieeffekt erreicht wurde, kann eine intraläsionale Applikation von Glukokortikosteroiden, zum Beispiel mit Triamcinolonacetonid, erfolgen.^49^

### Tacrolimus (off label)

Tacrolimus ist ein Calcineurininhibitor, der ausschließlich in Fallserienberichten erfolgreich eingesetzt wurde.^50^, ^51^, ^52^ Aufgrund des sehr guten Nebenwirkungsprofils kommt die topische Applikation von Tacrolimus als Zweit‐ oder Drittlinientherapie, gegebenenfalls unter Okklusion, in der Praxis häufig zum Einsatz.^4^, ^29^

### Licht‐ und Lasertherapien

#### UV‐Therapien

Zu den Therapien der NL mit ultravioletter (UV)‐Strahlung liegt die höchste dokumentierte Anzahl behandelter Patienten vor. Ein wesentlicher Vorteil der UV‐Therapien sind im Vergleich zu den Behandlungen mit Glukokortikosteroiden die verminderte atrophogene Potenz. Jedoch stellen der Zeitaufwand für die Patienten und die nicht (mehr) breite Verfügbarkeit in hautfachärztlichen Praxen ein Behandlungshindernis dar. Auch das mögliche, wenn auch seltene Auftreten von Plattenepithelkarzinomen in NL‐Herden muss bedacht werden.

Für die PUVA‐Therapie mit topischer oder systemischer Applikation des Photosensibilisators (meist 8‐Methoxypsoralen) und nachfolgender UV‐A‐Bestrahlung liegen die meisten Daten zur Anwendung bei NL vor. Diese Therapieform wird daher in der S1‐Leitlinie empfohlen. Sie wird bei NL vorwiegend als Creme‐ oder Bade‐PUVA durchgeführt. In einer Studie mit 30 mit Creme‐PUVA behandelten Patienten konnte eine Abheilung oder Verbesserung in gut der Hälfte der Patienten erreicht werden. Die Patienten mit fehlendem Ansprechen oder Verschlechterung hatten durchschnittlich eine geringere UV‐Dosis erhalten.^6^ Auch andere Autoren konnten eine Verbesserung oder Abheilung in der Mehrzahl der behandelten NL‐Fälle zeigen.^53^, ^54^ Auch Anwendung von UVA1 allein zeigte in bisher publizierten Studien bei 50%–66% der behandelten Patienten einen Nutzen,^55^, ^56^ so dass auch diese Therapieform erwogen werden kann.

### Andere physikalische Therapien

Bei der photodynamischen Therapie (PDT) muss bedacht werden, dass die Effekte durch die Eindringtiefe der Strahlenquelle und des Photosensibilisators (meist δ‐Aminolävulinsäure) begrenzt sind, was bei den tiefliegenden Entzündungsreaktionen der NL nachteilig ist. Dies könnte eine Erklärung sein, dass bei elf von 18 mit PDT behandelten Patienten keine Verbesserung der NL gezeigt werden konnte.^5^ Bei 65 NL‐Patienten konnte durch vorherige Kürettage eine Verbesserung der Wirkstoffpenetration erreicht und bei 66% der Fälle eine komplette Abheilung sowie bei 19% eine Reduktion der erkrankten Fläche um 80%–99% erreicht werden.^57^ Auch die Tageslicht‐PDT wurde bereits erfolgreich bei NL eingesetzt.^58^ Die PDT kann daher auch erwogen werden.

Eine 2020 erschienene Übersichtsarbeit betrachtete systematisch die vorliegende Evidenz verschiedener Publikationen zu Licht‐ und Lasertherapien.^59^ Hier zeigt sich die PUVA‐Therapie als effektivste Methode, gefolgt von PDT. Verfahren wie gepulste Lasertherapie, UVA1‐Therapie und CO_2_‐Laser wurden als schwächer wirksam bewertet.

### Kompressionstherapie

Die Kompressionstherapie der Unterschenkel ist traditionell eine Säule der konservativen Therapie der Patienten mit NL.^60^ Die diskutierten Ansatzpunkte sind hierbei Ödemreduktion, Verbesserung der Mikrozirkulation sowie antiinflammatorische Effekte. Zudem wurde die Komorbidität einer chronischen venösen Insuffizienz (CVI), die bei Patienten mit NL häufiger vorliegen soll, als potenzieller Trigger beschrieben.^61^ Die Kompressionstherapie ist eine wichtige und nebenwirkungsarme Behandlungsoption, die nach Ausschluss von Kontraindikationen bei allen Patienten mit NL an den unteren Extremitäten durchgeführt werden sollte.

### Operative Therapie

Es wurde mehrfach die erfolgreiche vollständige Exzision der Hautveränderungen bei NL und anschließende plastische Deckung mittels Hauttransplantat beschrieben.^61^, ^62^, ^63^, ^64^ Diese operativen Eingriffe wurden teilweise mit porcinen Materialien und/oder künstlich gezüchteten Hauttransplantaten durchgeführt. Aufgrund ihrer Invasivität bleiben diese Verfahren auf einzelne Läsionen der Necrobiosis lipoidica beschränkt.

### Wundtherapien bei ulzerierter Necrobiosis lipoidica

Für Patienten mit ulzerierter NL gibt es keine spezifische Wundtherapie.^65^ In diesen Fällen wird empfohlen, sich an den Phasen der Wundheilung zu orientieren und Faktoren wie Schmerzen, Exsudatmenge und lokale Infektionszeichen individuell zu berücksichtigen. Das M.O.I.S.T.‐Konzept bietet hierbei eine hilfreiche Orientierung.

### Systemische Therapien

Vor allem Patienten mit einer als schwer eingestuften NL‐Erkrankung und/oder Ulzerationen sollten systemisch behandelt werden. Die Therapieansätze zielen vornehmlich auf antiinflammatorische Wirkungen. Daher sollte zuerst eine Einschätzung der entzündlichen Aktivität der Hautveränderungen erfolgen. Bei fehlender entzündlicher Aktivität und Vorhandensein atropher, aber größenstabiler Restläsionen sind die Patienten darüber aufzuklären, dass keine Rückbildung der vorhandenen Hautveränderungen zu erwarten ist. Der Großteil der berichteten Effekte einer Systemtherapie basiert auf Fallberichten. Zur Einordnung der publizierten Daten fehlen zudem klare Beurteilungskriterien, was unter einer „Verbesserung“ der NL‐Läsionen unter Systemtherapeutika zu verstehen ist. Im Falle ulzerierter NL‐Läsionen wird die Abheilung der Ulzeration beschrieben.

### Systemische Glukokortikosteroide

Die Anwendung systemischer Glukokortikosteroide im entzündlichen Stadium einer NL findet weitgehenden Konsens in der klinischen Anwendung.^4^, ^29^ Es muss aber die Beeinflussung der diabetischen Stoffwechsellage beachtet werden.

### Fumarsäureester (*off label*)

Fumarsäureester besitzen eine Vielzahl immunmodulierender Eigenschaften mit Wirkung auf zahlreiche Gewebe‐ und Blutzellen wie Leukozyten, Keratinozyten aber auch Endothelien.^67^ Für Fumarsäureester liegen Daten einer prospektiven, nichtrandomisierten oder kontrollierten Studie vor.^68^ Achtzehn Patienten wurden in mit Fumarsäureester für mindestens 6 Monate systemisch behandelt. Der Therapieerfolg wurde klinisch, histologisch und mittels 20‐Mhz‐Ultraschall bewertet. Es konnte eine signifikante Verbesserung des klinischen Scores unter Therapie beobachtet werden. In der klinischen Praxis werden Fumarsäureester mit gutem Ansprechen bereits häufiger für NL‐Behandlung eingesetzt.^4^ Daher werden sie auch im Expertenkonsens empfohlen und als effektiv betrachtet.^29^

### Ciclosporin (*off label*)

Aufgrund der breiten Erfahrung mit der Anwendung des Calcineurininhibitors Ciclosporin in der Dermatologie kann die Anwendung in schweren Fällen von NL und Versagen von Erstlinientherapien empfohlen werden.^69^, ^70^

### Antimalariamittel (*off label*)

Zur Anwendung von Chloroquin und Hydroxychloroquin finden sich ebenfalls mehrere erfolgreiche Fallberichte in der Literatur.^70^, ^71^, ^72^ Eine Fallserie mit insgesamt acht Patienten zeigte eine signifikante Verbesserung in sieben Fällen.^73^ Aufgrund der kostengünstigen Therapie, der breiten Erfahrung in Anwendung und Therapieüberwachung kann eine systemische Behandlung mit (Hydroxy‐)chloroquin in Betracht gezogen werden.

### Biologika (*off label*)

Erfolgreiche Therapien mit den TNF‐α Inhibitoren Infliximab, Etanercept und Adalimumab bei Patienten mit NL wurde mehrfach beschrieben.^74^, ^75^, ^76^, ^77^ Meist waren es ulzerierte, therapierefraktäre NL‐Verläufe. Aufgrund der hohen Erfahrung in der Anwendung dieser Substanzen kann die Anwendung von TNF‐α Inhibitoren für schwere, therapierefraktäre NL‐Fälle empfohlen werden. Auch Biologika mit anderen Zielstrukturen wie Ustekinumab (anti‐IL12/IL‐23) wurden erfolgreich angewendet.^78^, ^79^ Ergebnisse einer Fallserie mit Secukinumab (anti‐IL‐17) in drei Fällen zeigten ein gutes Ansprechen.^80^

### Januskinase‐Inhibitoren (*off label*)

Es gibt verschiedene Januskinase (JAK)‐Inhibitoren, die bereits in Einzelfällen für die NL‐Therapie erfolgreich eingesetzt wurden.^81^, ^82^, ^83^, ^84^ Es zeigte sich bei einem Patienten, der aufgrund einer Polyzythämia vera mit dem JAK‐2 Inhibitor behandelt wurde, eine nebenbefundliche Besserung der NL. Eine 25‐jährige Patientin mit Typ‐1‐Diabetes und einer langjährigen NL‐Historie mit Ulzerationen und fehlendem Ansprechen auf diverse Therapien, wurde erfolgreich mit Tofacitinib behandelt. Eine Kombinationstherapie mit intraläsionalen Glukokortikosteroiden zeigte eine weitere Verbesserung des Befundes.

### Weitere systemische Therapieansätze (*off label*)

Obwohl Dapson bei anderen granulomatösen Erkrankungen erfolgreich angewendet wurde, existieren zur Anwendung bei NL kaum vielversprechende Daten. In einer Analyse von 98 NL‐Patienten wurde ein Viertel mit Dapson behandelt; in drei Fällen wurde ein gutes Ansprechen dokumentiert.^4^ In einer Expertenbefragung in Deutschland wurde von 43% der Dermatologen eine Empfehlung für die Therapie der NL mit Dapson ausgesprochen.^29^ Somit kann Dapson zumindest als Medikament der 2. oder 3. Wahl genannt werden.

Pentoxifyllin führt über antiinflammatorische Wirkungen zu einer Verbesserung der Mikrozirkulation. Zur Anwendung von Pentoxifyllin bei NL existieren einzelne Fallberichte,^85^, ^86^ sowohl von ulzerierten NL‐Fällen, als auch von NL mit ungewöhnlicher Lokalisation. Aufgrund der Datenlage kann ein Einsatz erwogen werden.

Eine Vielzahl von Substanzen wie Mycophenolatmofetil, Doxycyclin, Thalidomid, Clofazimin, Colchicin, Nicotinamid oder Tranilast wurden in Einzelfällen oder kleineren Fallserien erfolgreich zur Therapie der NL eingesetzt. Aufgrund der schwachen Datenlage kann für diese Substanzen keine Empfehlung ausgesprochen werden.

**ABBILDUNG 1:**
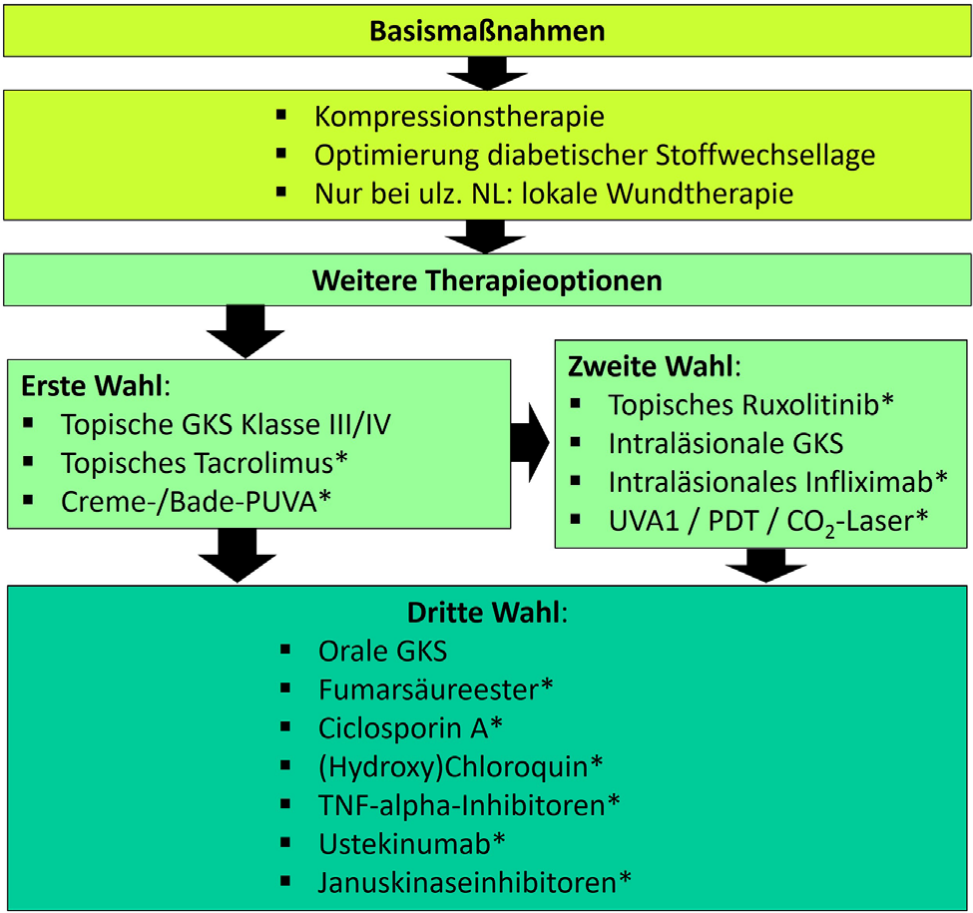
Ablaufbeschreibung der wichtigsten Therapie‐Empfehlungen der NL. *off label

Abbildung 1 fasst topische und systemische Therapieempfehlungen der Leitlinie zusammen.

### Besonderheiten bei Kindern und Jugendlichen

Berichte über NL bei Kindern und Jugendlichen liegen meist als Kasuistiken vor.^87^ Auffallend ist, dass fast alle betroffenen Kinder einen insuffizient eingestellten Typ‐1‐Diabetes zeigen. Therapeutisch wird man sich bei Minderjährigen vorrangig für topische Lokaltherapien wie hochpotente Glukokortikosteroide oder Calcineurininhibitoren entscheiden. Es gelten ansonsten für Kinder grundsätzlich die gleichen Empfehlungen für eine Abklärung assoziierter Erkrankungen wie für Erwachsene.

### Limitationen der Leitlinie

Randomisierte und kontrollierte klinische Studien zur Anwendung von Therapien bei NL fehlen bislang; die Zahl prospektiver Beobachtungsstudien ist ebenfalls gering. Es fehlen zudem eindeutige Kriterien zur Beurteilung der Verbesserung der NL‐Läsionen durch Therapie. Unter Berücksichtigung der aktuellen Literatur muss von einem Bias zu Gunsten eines positiven Therapieansprechens ausgegangen werden, da erfolgreiche Einzelfallberichte eher publiziert werden.^88^

### Fazit für die Praxis

Insgesamt betrachtet ist die wissenschaftliche Datenlage für die NL derzeit unzureichend und es besteht weiterer Forschungsbedarf. Es fällt zudem auf, dass bislang nur selten eine Unterscheidung zwischen ulzerierter und nicht‐ulzerierter Form der NL als Kriterium in den vorliegenden Studien angewandt wurde. Die Pathophysiologie der NL ist weiterhin ungeklärt. Hinweise aus effektiven Therapiemaßnahmen können hier die Grundlage für weitere Forschungsansätze sein. Da bei den NL‐Patienten oft ein hoher Leidensdruck besteht, ist es wichtig die wenigen wissenschaftlichen Erkenntnisse zu kennen und in entsprechenden Therapiekonzepte zu berücksichtigen.

## DANKSAGUNG

Open access Veröffentlichung ermöglicht und organisiert durch Projekt DEAL.

## INTERESSENKONFLIKT

Es wird auf die AWMF‐Langfassung der Leitlinie (AWMF‐Register‐Nr.: 013‐096, 2024) verwiesen.
